# Endovascular Embolization by Transcatheter Delivery of Particles: Past, Present, and Future

**DOI:** 10.3390/jfb8020012

**Published:** 2017-04-03

**Authors:** Rahul A. Sheth, Sharjeel Sabir, Savitri Krishnamurthy, Reginald K. Avery, Yu Shrike Zhang, Ali Khademhosseini, Rahmi Oklu

**Affiliations:** 1 Department of Interventional Radiology, Division of Diagnostic Imaging, MD Anderson Cancer Center, Houston, TX 77030, USA; rasheth@mdanderson.org (R.A.S.); shsabir@mdanderson.org (S.S.); 2 Department of Pathology, MD Anderson Cancer Center, Houston, TX 77030, USA; skrishna@mdanderson.org; 3 Wyss Institute for Biologically Inspired Engineering, Harvard University, Boston, MA 02115, USA; avery.reginald@gmail.com (R.K.A.); yszhang@mit.edu (Y.S.Z.); alik@bwh.harvard.edu (A.K.); 4 Biomaterials Innovation Research Center, Department of Medicine, Brigham and Women’s Hospital, Harvard Medical School, Boston, MA 02139, USA; 5 Division of Interventional Radiology, Mayo Clinic, 5777 E Mayo Blvd, Scottsdale, AZ 85054, USA

**Keywords:** biomaterials, biomedical devices, cardiovascular devices, drug delivery systems, microspheres, microfluidics

## Abstract

Minimally invasive techniques to occlude flow within blood vessels, initially pioneered in the 1970s with autologous materials and subsequently advanced with increasingly sophisticated engineered biomaterials, are routinely performed for a variety of medical conditions. Contemporary interventional radiologists have at their disposal a wide armamentarium of occlusive agents to treat a range of disease processes through a small incision in the skin. In this review, we provide a historical perspective on endovascular embolization tools, summarize the current state-of-the-art, and highlight burgeoning technologies that promise to advance the field in the near future.

## 1. Introduction

The past half-century has witnessed a tremendous expansion in the use of minimally invasive, endovascular techniques in medicine. Interventions predicated upon real-time image guidance to direct flexible catheters from an easily accessible, superficial blood vessel to a remote blood vessel deep within the body have revolutionized the clinical management of diseases involving almost every organ. A common paradigm in endovascular procedures is the performance of vascular embolization, a technique in which an occlusive agent is delivered through a catheter to obstruct flow within a targeted blood vessel. Embolization procedures are performed for a variety of medical conditions including to stop bleeding from a hemorrhagic stomach ulcer or to eradicate a tumor in the liver by blocking its blood supply. A diverse armamentarium of occlusive agents is currently clinically available, including metallic coils, injectable glues, and embolic particles. Each category of embolic agent is characterized by its respective strengths and weaknesses and enjoys several niche clinical scenarios for which it is ideally suited. Embolic particles were the first embolic agent developed and remain arguably the most versatile; moreover, they represent an ideal platform for tailored, highly localized transcatheter delivery of therapeutics. In this review, we summarize the current methods in the synthesis of particles and present the merits of the latest technologies such as microfluidic technology that have tremendous potential for advancing personalized particle design.

## 2. Historical Perspective on Particle Embolization Materials

Dr. Charles Dotter and his team at the Oregon Health Sciences University were the first to perform an embolization procedure in 1970. They used an autologous blood clot as the embolic agent to control a bleeding gastric ulcer by selective embolization of the right gastroepiploic artery in a critically ill patient. Soon after that historic day, autologous blood clot embolization was applied to other organs as well [1].

The preparation of fresh blood clots involves collecting fresh blood in a sterile container and allowing a clot to form. Once this occurs, the clot is cut into various sizes. By heating clots to 56–66 °C in a water bath, the lysis time is significantly prolonged. Similarly, the addition of aminocaproic acid to fresh blood inhibits plasmin, rendering the blood clot more resistant to lysis [2]. The clot can also be modified by adding cellulose, thrombin, or chronic clots to prolong the lysis time.

When injected, clots undergo fragmentation. This leads to distal migration, resulting in occlusion of vessels smaller than the injected clot. While this technique has faded into obsolescence, it is worth noting that autologous blood clots are almost completely biodegradable and cause a minimal vascular inflammatory response, two desirable features in certain clinical settings that not all contemporary embolization particles can claim.

A variation on autologous blood clots, autologous subcutaneous tissue and muscle have been used as embolization materials in the past as well [2]. The tissue is harvested from the patient immediately prior to embolization and then cut into small “meatballs” and suspended in saline. Unlike autologous blood clots, tissue is considered to be a permanent embolic agent, though there are very limited data on this technique [2].

The clinical need for permanent, radio-opaque embolic particles led to the evaluation of synthetic agents. Some of the earliest embolization particles were composed of lead shot [3] and Silastic [4], an inert silicone elastomer. The particle sizes were on the order of 1 mm and resulted in very proximal occlusion.

## 3. Modern Particle Embolization: Synthesis, Applications, and Limitations

### 3.1. Polyvinyl Alcohol Particles

Polyvinyl alcohol (PVA) is a biocompatible polymer that has been used in medicine since the 1950s. Early applications of PVA ranged from its use as a filling material following pneumonectomy to a skin substitute in burn patients [5].

PVA is highly compressible, and when it is dried while in compression, it will retain its compressed shape. When it is rehydrated, PVA will expand to up to 15 times its compressed size. PVA particles were first introduced as a transcatheter embolization tool in the 1970s [6]. PVA has since been used in a myriad of applications in Interventional Radiology, including embolization of arteriovenous malformations, mesenteric bleeding, primary malignancies, and bony metastases.

Upon injection, PVA particles adhere to the blood vessel wall, slowing flow and eventually leading to thrombus. PVA additionally induces an inflammatory response characterized by angionecrosis of the vessel wall. PVA is not biodegradable and is thus considered a permanent embolic agent. However, recanalization of embolized vessels with PVA can occur; this may be mediated by angiogenesis within the organized thrombus, resulting in resorption of thrombus. 

The fabrication of PVA particles for embolotherapy involves shaving or rasping the surface of a compressed block of PVA; the resultant pieces are sorted through sieves to separate the particles based on size. A drawback of this method is the heterogeneity in size and shape of the particles. A particle that is too large will lodge too proximally within a vessel, likely causing ineffective embolization. Alternatively, a particle that is too small will travel too distally, increasing the risk for end-organ damage. Examples of the latter from early formulations of PVA include facial nerve palsy after external carotid artery embolization [7] and bladder necrosis after pelvic embolization [8]. Moreover, PVA particles have a high coefficient of friction and can aggregate. This has two undesirable ramifications. First, the effective size of the particle increases significantly, leading to proximal rather than distal embolization. Second, the particles may “stick” within a microcatheter, impeding their injection into the patient.

### 3.2. Gelatin Sponge

**Figure 1:**
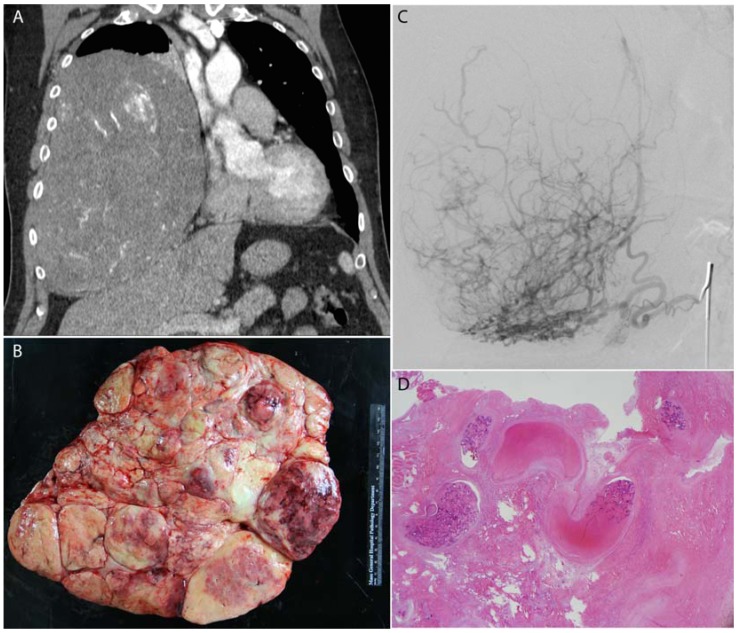
A large benign solitary fibrous tumor of the right pleura (**A**) was pre-operatively embolized with Gelfoam; (**B**) surgical resection photograph; (**C**) angiography of the right phrenic artery demonstrated exuberant vascularity within the mass; (**D**) histology demonstrates fragments of Gelfoam (acellular pink material) within blood vessels in the mass which is entirely infarcted.

Gelatin sponge is a non-antigenic carbohydrate prepared from purified skin gelatin. Gelatin sponge has a highly porous microscopic structure, allowing for absorption of up to 45 times its weight in fluid [9]. Like PVA, gelatin sponge has been used in medicine for over half a century, as it was initially introduced as a pro-coagulant for surgical patients in the 1940s. Also like PVA, gelatin sponge has been used as a transcatheter embolic agent for a broad range of clinical applications, including embolization of renal tumors and for emergent pelvic bleeding [6] (Figure 1). Gelatin sponge is commercially available as a powder or a sheet that can be cut to size; the powder form contains particles that are generally considered too small for clinical use, given the risk of ischemia by very distal embolization.

There are multiple techniques of preparing gelatin sponge for embolization [10]. Variability in technique and reliance on manual maneuvers such as cutting and pumping slurries between syringes results in a broad, unevenly distributed mixture of particle sizes that may range from less than 500 μm to above 2000 μm. This, in turn, limits reproducibility and predictability of where the embolic particles will lodge in the vascular tree.

Gelatin sponge incites a necrotizing arteritis and foreign body reaction that cause thrombus formation. The inflammatory changes persist for approximately 30–45 days, after which point gelatin sponge as well as the induced thrombus are no longer detectable.

While gelatin sponge is conventionally regarded as a “temporary” embolization material, there are limited data regarding the in vivo duration of occlusion following gelatin sponge embolization. In animal studies, the inflammatory reaction to gelatin sponge has been shown to subside by 4 months, without any trace of the material on pathology [11]. On the other hand, clinical cases of permanent embolization following gelatin sponge administration have also been described [12]. As such, complications such as ischemia can occur. For example, buttock ischemia and bladder gangrene have been reported following pelvic embolization with gelatin sponge [13]. Moreover, Gelatin sponge can retain air bubbles when not properly compressed, which can promote and sustain aerobic infectious agents and in theory lead to abscess formation [14]. The relationships between method of gelatin sponge preparation, volume of administration, or target organ on time course of embolization remain incompletely elucidated.

### 3.3. Microspheres

**Figure 2:**
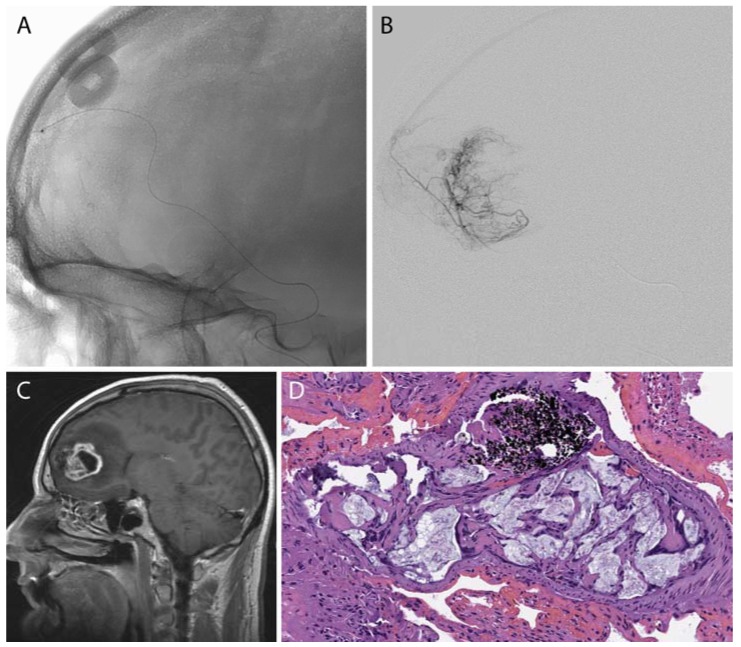
A frontal lobe glioblastoma multiforme was pre-operatively embolized using polyvinyl alcohol (PVA) microspheres. A microcatheter (**A**,**B**) was advanced to a branch of the middle meningeal artery that supplied the tumor (**C**). (**D**) Post-surgical histology demonstrated PVA (acellular light blue mucoid material) within the tumoral blood vessels in the tumor comprised of pleomorphic large tumor cells; of note, the black material represents the “liquid embolic” agent Onyx (Medtronic-Covidien, Irvine, CA, USA).

**Figure 3:**
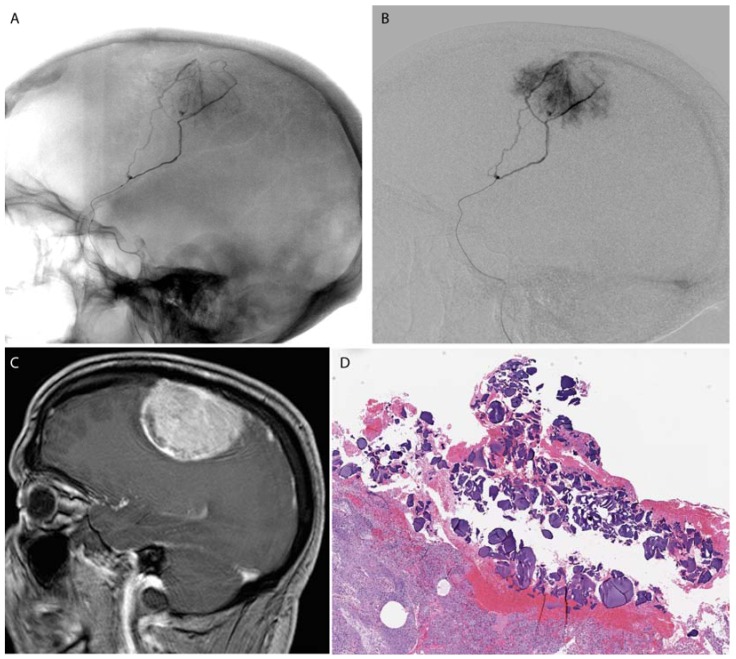
A falcine meningioma was pre-operatively embolized using PVA microspheres. A microcatheter (**A**,**B**) was advanced to a branch of the middle meningeal artery supplying the tumor (**C**). Post-surgical histology shows PVA microspheres within the tumoral tissue (**D**).

The development and clinical application of microspheres represents an important advancement in narrowing embolization particle size distribution (Figure 2 and Figure 3). Microspheres offer several advantages over PVA particles and gelatin sponge. As opposed to the irregular shape of PVA particles, the spherical geometry of microspheres decreases the risk of particle clumping within the microcatheter. Additionally, there is a strong correlation between the size of the microsphere and the diameter of the vessel at which occlusion occurs; the same cannot be said about PVA particles, as animal studies show poor correlation between the size of the occluded vessel and the size of the PVA particle.

Current microspheres commercially available in the United States are composed of PVA (Contour SE, Boston Scientific, Natick, MA, USA; Bead Block and LC Bead, Biocompatibles, Farnham, Surrey, UK), trisacryl-gelatin (Embosphere and EmboGold, Merit Medical Systems, South Jordan, UT, USA), polymethylmethacrylate with a coat of polyzene-F (Embozene, Celenova, San Antonio, TX, USA), and a super-absorbent copolymer (Quadrasphere, Merit Medical Systems, South Jordan, UT, USA). 

**Figure 4:**
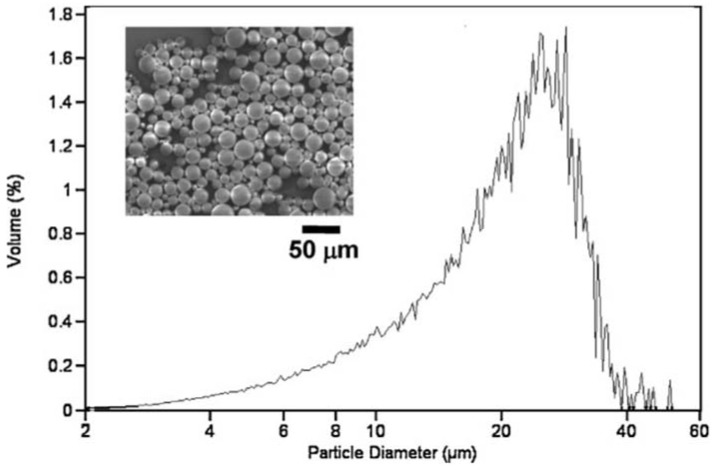
Scanning electron microscopy (insert) and size distribution of poly (lactic-*co*-glycolic acid) (PGLA) microparticles fabricated through conventional emulsion methods demonstrate a broad range of sizes even after filtering the particles through sieves. Reproduced with permission from [15].

For example, Embospheres are made of a cross-linked tris-acryl hydrophilic copolymer impregnated with gelatin; the role of gelatin is to facilitate endothelial adherence, thereby providing a more complete vascular occlusion. Microspheres are available in industry standard size ranges of 200 μm, approximately corresponding to the size of blood vessels accessible by microcatheters: 100–300 μm, 300–500 μm, 500–700 μm, 700–900 μm, and 900–1200 μm. Size sorting is achieved by filtering the synthesized particles through a series of sieves, and so for a given size range, one can expect that approximately 90%–95% of all microspheres are within the prescribed range. It is important to note as well that there is a Gaussian distribution of particle sizes within a given range (Figure 4).

Each commercial formulation demonstrates different compressibility, elasticity, and rigidity [16]. Even amongst microspheres composed of PVA, variations in their composition can have significant impacts on their function. For example, LC Beads do not aggregate inside blood vessels as other similarly sized PVA microspheres of differing compositions may, and as such LC Beads can result in deeper embolization. Additionally, Embosphere microspheres are much more rigid than Contour SE microspheres, which flatten with minimal resistance at low compression forces [17]. Moreover, the elastic recovery of Embosphere is much faster than Contour SE, which can require up to several seconds to recover [18]; this phenomenon is likely predicated upon the macroporous structure of Contour SE beads which allows for the extrusion of water from the microsphere upon compression, as opposed to the microporous structure of Embosphere and Bead Block which retains water. These properties play critical roles in determining the level of embolization. Given the deformability of Contour SE particles, it follows that these microspheres result in more distal occlusion than the less deformable Bead Block and Embosphere particles. That is, even within an identical size range, microspheres of different formulations will embolize vessels at different levels of the vascular tree. The pattern of embolization is of more than just academic interest; clinical evidence has demonstrated that Contour SE particles result in higher rates of partial devascularization of uterine fibroids compared Embosphere particles [19,20].

### 3.4. Drug Eluting Particles

**Figure 5:**
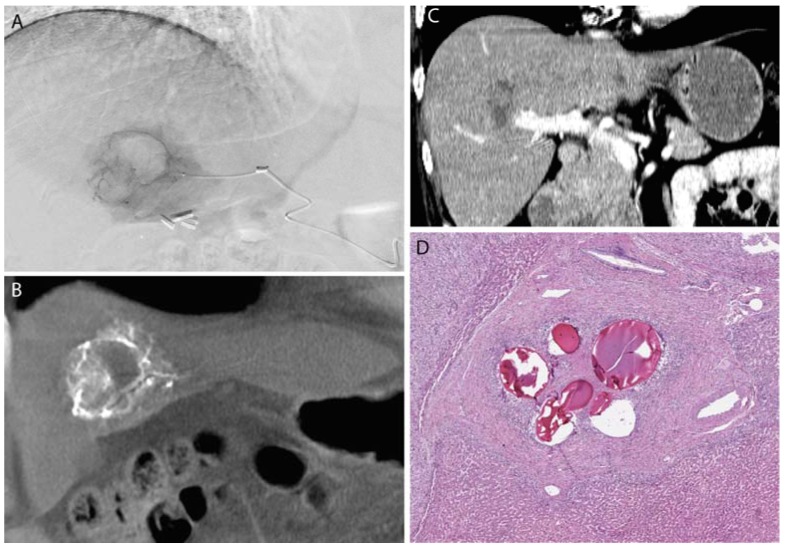
A leiomyosarcoma metastasis to the liver was embolized with 100–300 μm, doxorubicin-eluting microspheres to reduce tumor size and vascularity prior to surgical resection. Selective catheterization (**A**) of the tumor was performed, with proper positioning confirmed by cone-beam CT (**B**). Post-procedure CT (**C**) demonstrates no residual vascularity within the lesion. Following liver resection, microspheres were identified within the blood vessels in the tumor bed without any evidence of viable metastatic tumor (**D**).

**Figure 6:**
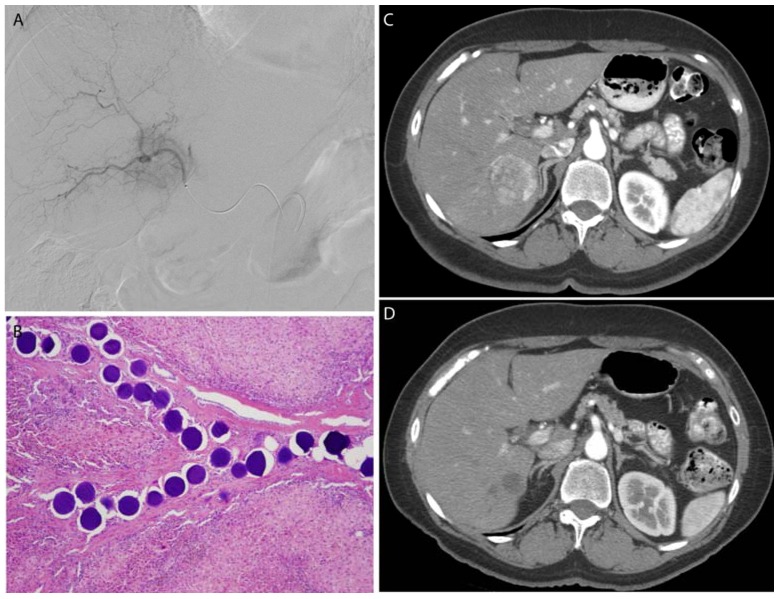
A breast cancer metastasis to the liver was embolized with 100–300 μm, doxorubicin-eluting microspheres to reduce tumor size and vascularity prior to surgical resection. Selective catheterization (**A**) of the tumor was performed, with delivery of the microspheres within the blood vessels of the tumor (**B**). Post-procedure CT (**D**) demonstrates complete response of the tumor as indicated by fibrosis alone without any evidence of residual tumor compared to the pre-procedure CT (**C**).

Drug eluting particles represent the next phase in the evolution of embolization particles (Figure 5 and Figure 6). The concept of controlled drug release is not a new one, having been first proposed in the 1960s. Formulations of pharmacologic agents that provide a sustained release of drugs over a protracted length of time, rather than through a single bolus dose, facilitate the diffusion of the drug through the interstitial space and improve its concentrations at the target. However, a persistent challenge for any sustained release system is its precise delivery to the target lesion, thereby minimizing the systemic toxicity and maximizing the therapeutic efficacy. Interventional Radiology has taken on this challenge, and over the past decade Interventional Radiologists have assumed an expanding role in the locoregional administration of therapeutics. This growth has been most evident in the realm of oncology through the advent of microspheres loaded with chemotherapeutic agents. These particles serve as a reservoir of chemotherapy, slowly releasing the drug over time. This results in improved penetration into the tumor as well as a decrease in systemic toxicity [21].

The composition and functionality of drug eluting particles can be broadly divided into two categories [22]. One class of drug eluting microspheres is composed of polymers that adsorb drugs through ionic interactions. The archetypical particle that exhibits this mechanism of drug loading is DC Bead (Biocompatibles, Farnham, Surrey, UK). DC Beads are commonly loaded with doxorubicin, and the controlled release of this drug is mediated by an ion-exchange mechanism, where a positively charged counter-ion (presumably Na^+^) binds to release doxorubicin from the inner surface of the bead. Not all pharmaceuticals are amenable to this method of drug loading, however, and so it cannot be generalized for all drugs. 

A second class of drug eluting microsphere is those that behave like sponges and “soak up” drugs in solution. Sodium acrylate polyvinyl (SAP) microspheres (Quadrasphere, Merit Medical Systems, South Jordan, UT, USA) are an example of this category: these particles absorb aqueous solutions to expand by up to 64 times their dry volume [22]. The limiting factor in drug loading for a SAP microsphere is the solubility of the therapeutic agent in saline. An important drawback to this absorption method of drug loading is that the mechanical characteristics of the particle, including size, rigidity, and elasticity, vary as a function of their drug loading. This can lead to unpredictability in the level of the vascular tree where embolization occurs.

## 4. Previewing the Future of Particle Embolization

### 4.1. Microfluidics

Since its inception in the 1940s, transcatheter embolization has enjoyed an increasingly sophisticated armamentarium of injectable particles. In considering what the future holds for this procedure, it is worthwhile to reflect upon the properties of conventional particles that show room for improvement. First and foremost, current embolic particles, including calibrated microspheres, are polydisperse. Bulk microparticle synthesis by emulsification followed by filtering with sieves yields particles with a non-uniform size distribution. This variability, coupled with differences in deformability due to particle composition, results in unpredictable depths of penetration and sites of embolization. Another concern with modern embolization particles is the risk of non-target embolization. This complication can occur despite appropriate operator technique and is doubly disconcerting due to the decreased delivery of particles to the target and risk of injury to nearby normal tissue. Finally, where the presently marketed embolization particles offer an impressive array of options in particle size, composition, and drug delivery, no two patients are the same, and relying on off-the-shelf products that work for most patients does not necessarily imply that they work for all patients.

Therefore, the principal characteristics of an ideal embolization particle include a monodisperse size distribution, specific activation within the target lesion to diminish the risk of non-target embolization, and a customizable design that can be tailored to patients on an individual basis. While these features would have been considered unfeasible as recently as the 1990s, innovations in micro-fabrication are narrowing the gap between theory and reality.

**Figure 7:**
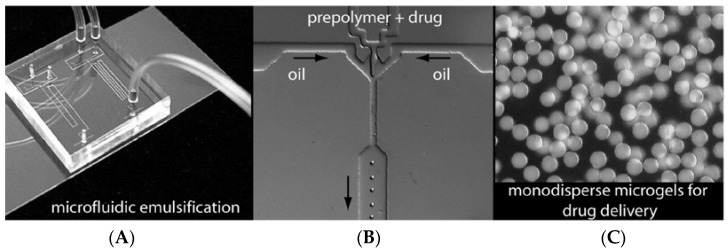
(**A**) Optical image of a microfluidic chip featuring two parallel droplet formulators. (**B**) Optical image of an in-line droplet generating nozzle for particle formulation and emulsification. (**C**) Optical image of a monodisperse population of 10 μm diameter microspheres. Reproduced with permission from [23]. Copyright (2005) American Chemical Society.

One such envelope-pushing technology that is on the cusp of clinical translation is microfluidics. Microfluidics is a discipline that resides at the convergence of physics, chemistry, and bioengineering. It is the study of the behavior and manipulation of geometrically constrained fluids at a sub-millimeter scale. Microfluidics systems have been investigated in the development of biodegradable, drug-bearing microspheres, with remarkable pre-clinical success (Figure 7).

**Figure 8:**
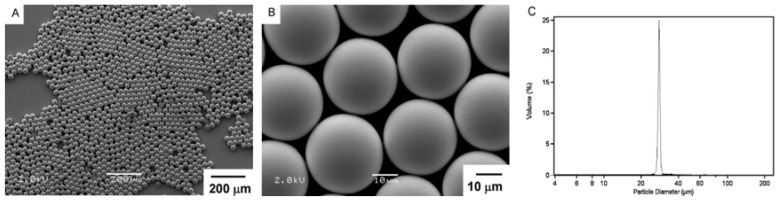
Microfluidics systems create monodisperse populations of microspheres. (**A**,**B**), scanning electron microscopy shows microspheres produced by microfluidics that are spherical and uniform in size. (**C**), size distribution chart shows a near “delta” function of particle size, consistent with a monodisperse population. Reproduced with permission from [15].

Designing embolization particles by microfluidics affords numerous advantages over conventional fabrication techniques. Microfluidic systems have small footprints, are low cost, and require minimal training to operate. They are highly tunable systems that allow a user to specify on demand the size of microsphere generated by the system. The output from microfluidics systems is highly monodisperse microspheres (Figure 8). These features open the door to personalized particle design and synthesis that can be performed at the point of care.

Moreover, given that every particle produced in a microfluidic system is the same size, no size filtering of the generated particles is required. This results in much higher yields than standard microsphere synthesis methods, in which size specificity is achieved by removing the large particles through a series of filters. This can play a key role in reducing the cost of microsphere fabrication, particularly if they are loaded with an expensive therapeutic agent.

Homogenous particle morphology also assists in predicting drug delivery in drug-eluting particles. There are several critical parameters that impact the spatial and temporal kinetics of drug release from microspheres. These include the rate of degradation of the microsphere, the internal architecture of the microsphere, the solubility of the therapeutic, and the concentration of drug on the microsphere. By standardizing the mechanical properties of all particles delivered to the patient, the rate of drug delivery can be estimated with high fidelity, reproducibility, and consistency. Monodisperse microspheres demonstrate slower overall rates of release compared to conventionally fabricated polydisperse microspheres. Furthermore, larger particles release drugs at a more gradual rate than smaller particles. Given the narrow distribution in particle size generated by microfluidic devices, interventional radiologists could seek to optimize delivery kinetics by using particles just at the size limit of the desired level of embolization, as the risk of administering particles larger than desired is essentially non-existent.

Microfluidics systems allow for flexibility in the material composition of microspheres. Most drug-eluting particles are composed of a hydrogel, which is a three-dimensional hydrophilic polymer that can absorb a large amount of water without losing its spherical shape. There exists a vast array of hydrogels, including those that respond to physical or chemical stimuli such as heat, pH, temperature, and glucose [24]. For example, thermosensitive poly (organophosphazene) hydrogels provide a sustained release of doxorubicin; one can easily envision the synergy of such a drug-eluting microsphere with percutaneous thermal ablation methods for the treatment of hepatic malignancies. Thermosensitive polymers have already penetrated the endovascular surgery market and have been used as temporary vascular occlusion devices during arterial bypass procedures [25]. In addition to pharmacologic agents, microspheres can be loaded with biological agents through microfluidics techniques. For example, microparticles can be loaded with viral vectors which can deliver targeted gene therapy [26].

### 4.2. Tissue Engineering

Tissue engineering, like microfluidics, is another well-developed field in bioengineering that can contribute to the improvement of particle embolization materials. Tissue engineering at its core is the control of tissue development in vitro and in vivo. Materials previously used for particle embolization can be redesigned with tissue engineering in mind to improve biocompatibility and functionality.

Incorporating tissue engineering into the design of embolization particles provides a framework from which biochemical factors, through small molecule encapsulation or biomaterial design, or cells can be delivered to actively impact the local environment surrounding an occluded vessel. The tissue engineered particles can be coated with extracellular matrix proteins such as collagen, fibronectin, or laminin to improve the endothelialization of embolic particles [27]. Bioactive molecules such as tissue plasminogen activator (tPA) or aminocaproic acid can also be encapsulated in chitosan or poly(2-hydroxyethyl methacrylate) to impact coagulation surrounding the embolic particle [28,29]. More active enzymes such as thrombin have also been encapsulated in synthetic polymer microspheres to accelerate clotting at the site of embolism [30].

## 5. Conclusions

Particles for endovascular embolization have evolved from materials such as lead pellets, muscle, fat, autologous blood clot and gelatin sponge to particles comprised of complex polymers with varying biochemical properties which can be modified to carry therapeutic payloads. Currently available particles span a broad spectrum of sizes, shapes, and materials and compose a versatile toolkit applicable to a myriad of disease processes. This toolkit, and by extension the clinical utility and applicability, is poised to expand substantially through emerging technologies that may revolutionize the accuracy and efficacy of particles.
